# Consensus on Use of the Term “App” Versus “Application” for Reporting of mHealth Research

**DOI:** 10.2196/jmir.3460

**Published:** 2014-07-17

**Authors:** Thomas Lorchan Lewis, Matthew Alexander Boissaud-Cooke, Timothy Dy Aungst, Gunther Eysenbach

**Affiliations:** ^1^Warwick Medical SchoolUniversity of WarwickCoventryUnited Kingdom; ^2^Department of PharmacyMCPHS UniversityWorcester, MAUnited States; ^3^JMIR PublicationsToronto, ONCanada; ^4^University Health Network, Techna Institute, and University of TorontoToronto, ONCanada

**Keywords:** medical app: mobile app, medical informatics: smartphone, mHealth

## Letter

We read with great interest a number of recent articles from the Journal of Medical Internet Research (JMIR) published by Bierbrier et al [1], Kim et al [2], and Choi et al [3], investigating different aspects of mHealth technology, specifically medical “applications” or “apps”. Many individuals, researchers, academic institutions, and other professional bodies often use these terms interchangeably. It is apparent that there is no clear consensus for which term should be used as both are recognized terms for a software program designed to run on smartphones, tablet computers and other mobile devices.

**Figure 1:**
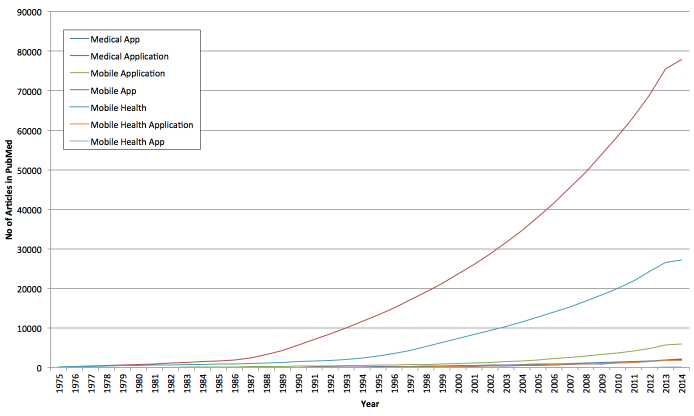
A graph showing cumulative number of PubMed search results by year since 1975 for keywords related to mobile applications (search carried out on April 7, 2014).

As the field continues to expand, we believe that the inconsistent use of terminology used may present a problem for future researchers to systematically identify and conduct appropriate literature searches. Figure 1 shows a graph of the cumulative number of PubMed search results by year related to keywords relating to “medical application” and “app” respectively since 1975 (Figure 1). This shows the clear exponential growth in this field as the amount of research in this field continues to grow. In particular it is worth drawing attention to the fact that the term “medical application” is used considerably more often in a number of medical specialties indicating it is not specific to mobile heath. The inconsistent use of terminology is also apparent in the use of keywords and Medical Subject Headings (MeSH) terms. It is currently unclear which are the most appropriate keywords for selection with many researchers using a variety of terms, common examples include: mobile device vs smartphone vs cellphone, mobile tablet vs mobile computer, and applications vs apps.

We believe it is now time for the mHealth research community to come to a universal consensus on whether studies should refer to medical “apps” or “applications”. Standardization of terminology will enable researchers and other health care professionals to:

- identify relevant articles and improve the literature search process through use of common search terms across different modalities;
- identify common MeSH terms which describe interventions utilizing mobile medical software (which is currently lacking);
- ensure databases categorize mobile health interventions more effectively for future researchers;
- improve the distinction between software designed for use on mobile devices and desktop devices;
- improve reporting of studies investigating mobile health interventions.

We believe that we should use the term “app” [plural “apps”], rather than “applications” for the following reasons:

1. this is the commonly used term in the public domain, social media and by the major hardware manufacturers;
2. the term medical application is not specific enough to software mHealth programs utilized on mobile devices. Many other medical research fields utilize the term “medical applications” whilst the term “app” is more specific to software programs utilized on mobile devices;
3. there is currently no MeSH keyword for the term “mobile medical app” while there are already preexisting MESH keywords related to “medical informatics application” or “mobile app”, neither of which is specific for mHealth interventions;
4. use of the term “application” may be misleading particularly for lay users who may believe this term represents software designed for desktop computers.

In conclusion, we would recommend that leading eHealth medical informatics publications such as the JMIR journals implement a policy to utilize common nomenclature moving forward to facilitate improved reporting of studies investigating mobile medical app interventions.

*Thomas Lorchan Lewis, Matthew Alexander Boissaud-Cooke, Timothy Dy Aungst*

## Editorial Response

We appreciate the thoughtful and important comment of Lewis et al and fully agree about the need of a consistent terminology for mobile apps, as well as the preferential use of the term “app”. In fact, JMIR Publications maintains an internal style guide, and already in June 2013 introduced a new guideline for our copyeditors where we explicitly ask to enforce use of the word “app” rather than “application”, even though the word “app” was originally a short form of “application software”. The use of the word “application” in the title of the paper cited by Lewis et al [2] was an oversight on the part of the freelance copyeditor assigned to the manuscript, and we will be more vigilant in the future to enforce the term “app”. We also have other standards which should help indexing and retrieval in particular in the context of systematic reviews. For example, we prefer the term “mobile phone” over “smartphone” in title and abstract, as the latter is often forgotten by systematic reviewers searching for “mobile” technology studies (we also noted it is not mentioned in Figure 1). In addition, all papers referring to mobile technologies are indexed with the theme keyword “mhealth”. These policies extend to all JMIR journals, including Journal of Medical Internet Research, JMIR Research Protocols, JMIR mHealth and uHealth, JMIR Serious Games, JMIR Medical Informatics, JMIR Human Factors, JMIR Mental Health, interactive Journal of Medical Research, Medicine 2.0 and others. We hope that other journals will follow and adopt these terminology standards, which should ultimately also make it into reporting guidelines such as CONSORT EHEALTH [4] .

*G. Eysenbach, Editorial Director, JMIR Publications*

## Abbreviations

MeSH: Medical Subject Headings
